# Multimodal strategy in surgical site infections control and prevention in orthopaedic patients – a 10-year retrospective observational study at a Polish hospital

**DOI:** 10.1186/s13756-020-0680-6

**Published:** 2020-01-23

**Authors:** Małgorzata Kołpa, Roża Słowik, Marta Wałaszek, Zdzisław Wolak, Anna Różańska, Jadwiga Wójkowska-Mach

**Affiliations:** 1 State Higher Vocational School in Tarnów, Szpital Wojewódzki im. Św. Łukasza, Tarnów, Poland; 2 Department of Microbiology, Polish Society of Hospital Infections, Jagiellonian University, 18 Czysta St., 31-121 Kraków, Poland

**Keywords:** Deep incisional surgical site infections, Hip prosthesis, Knee prosthesis, Open reduction of fracture, Closed reduction of fracture with internal fixation, Infection control and prevention

## Abstract

**Introduction:**

Surgical site infections (SSIs) are among the most common healthcare-associated infections. They are associated with longer post-operative hospital stays, additional surgical procedures, risk of treatment in intensive care units and higher mortality.

**Material and methods:**

SSIs were detected in patients hospitalized in a 40-bed orthopaedics ward in 2009–2018. The total number of study patients was 15,678. The results were divided into two 5-year periods before and after the introduction of the SSI prevention plan. The study was conducted as part of a national Healthcare-Associated Infections Surveillance Programme, following the methodology recommended by the HAI-Net, European Centre for Disease Prevention and Control Program (ECDC).

**Results:**

One hundred sixty eight SSIs were detected in total, including 163 deep SSIs (SSI-D). The total SSI incidence rate was 1.1%, but in hip prosthesis: 1.2%, in knee prosthesis: 1.3%, for open reduction of fracture (FX): 1.3%, for close reduction of fracture (CR): 1.5, and 0.8% for other procedures. 64% of SSI-D cases required rehospitalisation. A significantly reduction in incidence was found only after fracture reductions: FX and CR, respectively 2.1% vs. 0.7% (OR 3.1 95%CI 1.4–6.6, *p* < 0.01) and 2.1 vs. 0.8% (OR 2.4 95%CI 1.0–5.9, *p* < 0.05). SSI-Ds were usually caused by Gram-positive cocci, specially *Staphylococcus aureus*, 74 (45.7%); Enterobacteriaceae bacillis accounted for 14.1% and Gram-negative non-fermenting rods for 8.5%.

**Conclusions:**

The implemented SSI prevention plan demonstrated a significant decrease from 2.1 to 0.7% in SSI-D incidence only in fracture reductions, without changes in epidemiology SSI incidence rates in other procedures. Depending on the epidemiological situation in the ward, it is worthwhile to surveillance of SSIs associated to different types of orthopaedic surgery to assess the risks of SSI and take preventive measures.

## Introduction

In the European Union, there is a great diversity with regard in the practices of control and employment of staff for infection control teams. In many countries, infection surveillance programmes struggle with human resource shortages and strong local cultural conditions [1]. Poland, located in Central and Eastern Europe (CEE), began to implement a system of surveillance of healthcare-associated infections (HAIs) after the political transformation of 1989. Prior to that, there were no organizational structures or mechanisms allowing to detect, qualify or prevent HAIs; and any attempts to start debating this issue in the public sphere ended in failure. Legal regulations providing the foundations for an infection control system did not come into existence until 2001. Therefore, in Poland, the HAI surveillance system is relatively young, and, given no tradition of HAI control and a deeply rooted uncertainty regarding this field, the conditions are not favourable for a continuous, active surveillance or even to registration of HAIs. It is also substantiated by Allerberger et al. [2] who state that, in CEE, as well as publicly available information on epidemiological methods and indicators, are often insufficient. This is corroborated by Ider et al. [3] in their study of how infection control systems function in the former Soviet bloc. They found weak commitment, lack of resources, poor specialist knowledge and insufficient reporting or publishing of information on HAI epidemiology. Additionally, the CEE countries exhibit enormous differences with regard to legislation, structural elements and indicators of the methods for infection control and prevention [2].

Also, according to a report on HAIs by WHO (for 1995–2010), in low- and middle-income countries, regular HAI surveillance can prove difficult. In the report, our region, i.e. Central and Eastern Europe, is represented only by Lithuania and Latvia, as well as Serbia (which is not part of the EU) [4]. In Poland, there are no clear or straightforward rules of conduct in surveillance and there is no obligation to provide information on infections to the public, hence, the data on Polish hospitals are scarce, which may confirm the thesis put forward by the authors of the WHO report on difficulties in implementing HAI surveillance.

Zingg et al. have identified ten key elements essential for an effective infection control in the day-to-day practice of every hospital. They are, among others, organization of infection control structures at the hospital level; staff: nurses’ workload and forms of employment; correct application of guidelines; education and practice; multimodal and multidisciplinary prevention programmes, positive organizational culture and audit and feedback [5]. Hence, a meaningful impulse for the adoption of activities associated with infection control can arise in the form of the hospital’s efforts to carry out accreditation, done by an external entity. In Poland, accreditation is voluntary and free of charge and carried out by a unit run by the Ministry of Health.

The objective of this study was to analyse the impact of infection control and prevention activities in patients with locomotive organ diseases treated surgically and the implementation of “perioperative control card (perioperative checklist)” which was part of the hospital’s preparation for accreditation. The authors and the Infection Control Team (ICT) of the investigated hospital, in their previous analysis of research material from 2009 to 2013 [6], indicated a problem of surprisingly high SSI incidence rate in surgical patients and the urgent need for action concerning the prevention and control of SSIs. In 2014, the hospital was preparing, for the first time, to undergo the process of accreditation and the ICT decided to, at the same time, implement a series of interventions that should significantly improve patient safety; hence, the analysed material was divided into two time periods: years 2009–2013 (before the accreditation) and years 2014–2018 (after the accreditation).

## Material and methods

The investigated trauma and orthopaedics ward has 40 beds and is located in southern Poland. The hospital has its own microbiological laboratory. In the hospital, since 2001, there has been an active Infection Control Team which consists of a doctor, who is employed on 1/3 full-time equivalent basis, and 4 full-time nurses. Active surveillance of surgical site infections was introduced in the examined department in 2008.

In compliance with The International Classification of Diseases, Ninth Revision, Clinical Modification (ICD-9-CM) and with the methodology of the National Healthcare Safety Network (NHSN), the operations performed have been divided into: hip arthroplasties (HPRO), knee arthroplasties (KPRO), and other musculoskeletal surgeries: open (FX) and closed (CR) reduction of fracture, knee arthroscopy (KART) and removal of fixation device (UZ) (Table 1). Patients are admitted both electively and emergently. In the study period, emergencies constituted 42.8% of CRs, 30.1% of FXs, 8.0% of UZs, 3.9% of ARTKs, 2.9% of HPROs, 1.3% of KPROs, and 2.3% of others. A single dose of cephazolin was applied in preoperative antibiotic prophylaxis at a dose of 1 or 2 g i.v. (according to body mass) in the operating room. For HPRO and KPRO, prophylaxis with cephazolin was continued every 8 h for 24 h.

**Table 1:** The list of surgeries and ICD-9 codes

Code	Operative procedures: ICD-9
HPRO	Hip Prosthesis (HPRO): 00.70–00.73; 00.85–00.87; 81.51–81.53.
KPRO	Knee Prosthesis (KPRO): 00.80–00.84; 81.54; 81.55.
FX	Open Reduction of Fracture (FX): 79.21; 79.22; 79.25; 79.26; 79.31; 79.32; 79.35; 79.36; 79.51; 79.52; 79.55; 79.56.
CR	Closed Reduction of Fracture with Internal Fixation (CR): 79.11–79.18; 79.191–79.194
UZ	Removal of Fixation Device (UZ): 78.6
KART	Knee Arthroscopy (KART): 80.26
OTHER	Orthopaedic surgery other than HPRO, KPRO, FX, CR, ZU, KART

The hospital in which the research was carried out participates in a voluntary nationwide system of active HAIs monitoring, in accordance with the methodology of the Healthcare-Associated Infections Surveillance Network (HAI-Net), European Centre for Disease Prevention and Control (ECDC) [7]. In 2008–2012, infections were detected, qualified and registered according to the definitions by the National Healthcare Safety Network (NHSN) [8], which are in line with the ECDC definitions [7, 9]. The SSIs were qualified as superficial / deep incisional or organ/space. The follow-up period was 30 days for the superficial SSIs, and 90 days for deep or organ/space infections following arthroplasties. Due to the predominance of deep incisional SSIs (SSI-D), the authors have decided that only this group will undergo detailed analysis. The postoperative follow-up visits take place 6 weeks after HPRO and KPRO, in other cases: 2 or 3 days after discharge.

The results of the observation were divided into two time periods: years 2009–2013 (first period) and years 2014–2018 (second period). In 2013, works have commenced to prepare the hospital and the examined department for the process of accreditation. In the framework of this development, in 2014, a perioperative checklist was introduced in the ward under investigation, which inspected the execution of procedures, including the newly-implemented practices, among others:

1. bathing the patient prior to surgery with antiseptic soap,
2. changing bed linen and the patient’s underwear immediately before surgery,
3. operative field hair removal immediately before surgery using surgical clippers – without the use of blades,
4. surgical hand hygiene according to the WHO recommendations,
5. disposable surgical draping,
6. the application of antiseptic to the edges of the wound before stitching the skin,
7. giving systematic (every 6 months) feedback concerning the epidemiology and microbiology of infections.

The ward was granted a positive Accreditation Certificate in 2014, thus, this year became the basis for the comparison of the results from the period before and after accreditation.

To compare both periods, the choice was made to examine the SSI incidence rate and analyse variables, such as: waiting time for surgery in the ward, duration of stay in the ward, number of days from surgery to SSI detection, the number of SSIs detected after discharge. Additionally, demographics of surgical patients, their age and genders were also provided. Incidence rate was calculated as the number of SSI cases per 100 operations.

Statistical analysis of the collected material employed the IBM SPSS (SPSS – *Statistical Package for the Social Sciences*) STATISTICS 24 (Armonk, NY, USA) and Microsoft Excel (Microsoft Office 2016, Redmond, WA, USA) software. Statistical analysis was carried out with the use of basic statistical parameters, i.e. mean, 95% confidence intervals for the mean, median, and standard deviation. The risk of developing SSI-D in particular types of surgeries was compared for the two analysed periods by calculating the odds ratio (OR). In order to compare the frequency of occurrence of the variants of the qualitative trait, Pearson’s chi-square test of independence was used, Fisher’s exact test was employed for variables of small numbers and the ANOVA test for quantitative variables. The level of significance was *p* < 0.05. The use of data was approved by the Bioethical Committee of the Jagiellonian University (No. KBET/122.6120.118.2016 from May 25, 2016). All the data entered into the electronic database and analyzed in this study were previously anonymized.

Our experiences from the previous years have already been partly discussed, but those discussions concerned different types of infections and patient populations [6].

## Results

The overall number of subjects included in the study, during the entire 10-year period, amounted to 15,678 surgical patients. In total, 272 different HAIs were detected in various types of procedures, of which 168 (62%) were SSIs, including 163 SSI-D, i.e. 98% of all SSIs. HAI incidence rate amounted to 1.7%, SSI incidence was 1.1% (SSI-D 1%). The first symptoms of SSI-D were generally observed 37 days after surgery (95% CI 29.8–44.9), and the majority of SSI-D cases, i.e. One hundred four people (64.2%), required rehospitalisation – the diagnosis of SII-D was made after the patient was discharged from hospital. In the analysis of differences between the two periods studied, no statistical differences were found in SSI-D incidence in HPRO (*p* = 0.740), KPRO (*p* = 0.068) and in KART (*p* = 0.232) and UZ (*p* = 0.530) procedures. A significantly lower incidence was found after fracture reductions: FX (27 before and 10 cases of SSI-D after intervention) and CR (19 before and 6 cases of SSI-D after intervention), SSI-D incidence were respectively 2.1% vs. 0.7% (OR 3.1 95%CI 1.4–6.6, *p* < 0.01) and 2.1 vs. 0.8% (OR 2.4 95%CI 1.0–5.9, *p* < 0.05). Among the other elements under investigation, the patient age changed significantly: in HPRO, it increased from the initial 67 years to 70 years and, in KART, it decreased from 35 years to 33 years. There was a significant increase concerning the proportion of men in HPRO, from 35.3 to 41.9%, and in KART, from 61.9 to 69.8%. The organization of work in the ward expressed in duration of hospitalization have changed significantly, especially for HPRO, but the extent of changes was small: 1 day for HPRO, from 13 days before to 12 days after intervention, 2 days for fracture reduction from 10 and 8 days before to respectively 8 and 6 days after intervention, and also 2 days for KART, from 5 days before to 3 days after intervention (Table 2).

**Table 2:** Incidence rate of deep incisional surgical site infections (SSI-D) considering age of patients, waiting time for surgery in days, duration of stay in the ward in days, number of days from surgery to SSI detection, detection before or after discharge from hospital, gender of patients in HPRO and KPRO endoprosthesis surgery in 2009–2013 vs 2014–2018

Operation type	Endoprosthesis				Fracture reduction				Knee arthroscopy, removal of fixation device and others			
	HPRO		KPRO		FX		CR		KART		UZ	
Year	2009–2013	2014–2018	2009–2013	2014–2018	2009–2013	2014–2018	2009–2013	2014–2018	2009–2013	2014–2018	2009–2013	2014–2018
Number of surgeries	1491	1224	347	500	1303	1356	898	724	637	669	655	588
Number of SSI-D	16	16	4	9	27	10	19	6	6	3	5	5
Incidence rate (%)	1,1	1,3	1,2	1,8	2,1	0,7	2,1	0,8	0,9	0,4	0,8	0,9
OR (95%CI)	1.2 (0.61 to 2.45)		1.6 (0.48 to 5.15)		0.4 (0.17 to 0.73)		0.4 (0.15 to 0.97)		0.5 (0.12 to 1.90)		1.1 (0.32 to 3.87)	
Fisher’s test; OR (95%CI)	*p* = 0.740;		*p* = 0.068;		*p* < 0.01;		*p* < 0.05;		*p* = 0.232; 2.1 (0.5–8.4)		*p* = 0.530; 0.9 (0.3–3.2)	
Waiting time for operation in the ward [days]												
Median (Me); SD	3 (2); 4,1	3 (2); 4,5	2 (2); 2,6	3 (2); 3,7	3 (2); 4,1	3 (2); 4,3	2 (1); 3,6	2 (1); 2,8	2 (1); 2,0	1 (1); 1,2	2 (1); 5,1	2 (1); 3,9
ANOVA (p)	*p* = 0,049		*p* = 0.383		*p* = 0.641		*p* = 0,048		*p* < 0.05		*p* = 0.117	
Duration of stay in the ward [days]												
Median (Me); SD	13 (11); 6,6	12 (10); 7,5	13 (10); 7,3	13 (10); 8,2	10 (8); 8,6	8 (6); 7,3	8 (7); 6,8	6 (5); 5,2	4 (3); 3,4	4 (3); 2,4	5 (3); 9,0	3 (2); 4,9
ANOVA (p)	*p* = 0,047		*p* = 0.543		*p* < 0.001		*p* < 0.001		*p* = 0.186		*p* < 0.001	
Number of days from surgery to SSI detection												
Median (Me); SD	47 (45); 36,1	43 (12); 73,9	25 (15); 20,0	52 (25); 88,3	41 (17); 47,9	50 (29); 46,3	61 (44); 57,4	34 (24); 31,3	26 (11); 31,6	24 (24); 5,5	9 (3); 14,3	11 (4); 17,0
ANOVA (p)	*p* = 0.840		*p* = 0.627		*p* = 0.617		*p* = 0.293		*p* = 0.919		*p* = 0.876	
SSI-D: diagnosis mode [n]												
Before discharge	5 (31,3)	8 (50,0)	1 (25,0)	4 (44,4)	12 (46,2)	1 (11,1)	2 (10,5)	3 (50,0)	2 (33,3)	0 (0,0)	3 (60,0)	2 (40,0)
After discharge	11 (68,7)	8 (50,0)	3 (75,0)	5 (55,6)	14 (53,8)	8 (88,9)	17 (89,5)	3 (50,0)	4 (66,7)	3 (100)	2 (40,0)	3 (60,0)
Fisher’s test; OR (95%CI)	*p* = 0.238; 0.5 (0.11–1.92)		*p* = 0.636; 0.4 (0.03–5.71)		*p* = 0.066; 6.9 (0.75–62.96)		*p* = 0.070; 0.1 (0.01–1.03)		*p* = 0.417; n/a		*p* = 0.50; 2.3 (0.18–28.26)	
Patient age [years]												
Median (Me); SD	67 (67); 12,3	70 (71); 11,8	69 (71); 8,8	69 (70); 8,1	60 (61); 21,7	61 (63); 21,5	54 (58); 22,1	55 (59); 21,2	35 (34); 13,0	33 (31); 12,9	46 (44); 22,3	44 (43); 18,8
ANOVA (p)	*p* < 0.001		*p* = 0.538		*p* = 0.212		*p* = 0.507		*p* < 0.01		*p* = 0.183	
Gender												
Female	975 (64,7)	721 (58,1)	247 (71,2)	374 (74,8)	749 (56,3)	752 (55,1)	435 (47,4)	325 (44,5)	245 (38,1)	203 (30,2)	277 (40,7)	223 (37,9)
Male	532 (35,3)	519 (41,9)	100 (28,8)	126 (25,2)	581 (43,7)	614 (44,9)	482 (52,6)	405 (55,5)	398 (61,9)	469 (69,8)	404 (59,3)	365 (62,1)
Fisher’s test; OR (95%CI)	*p* < 0.001; 1.3 (1.13–1.54)		*p* = 0.082; 0.8 (0.61–1.13)		*p* = 0.267; 1.1 (0.90–1.23)		*p* = 0.129; 1.1 (0.93–1.37)		*p* < 0.01; 1.4 (1.13–1.79)		*p* = 0.173; 1.1 (0.89–1.41)	

*HPRO* Hip Prosthesis, *KPRO* Knee Prosthesis, *FX* Open Reduction of Fracture, *CR* Closed Reduction of Fracture with Internal Fixation, *KART* knee arthroscopy, *UZ* removal of fixation device, *OR* Odds Ratio, *SSI-D* deep incisional surgical site infections, *SD* standard deviation, *Me* median, *n/a* not available;

Among the SSI-D observed, 10.4% of the cases were not diagnosed microbiologically: no material for microbiological testing was collected in 9 cases, the aetiological factor could not be isolated from the materials (wound swabs and/or blood) in 8 cases. The remaining cases were dominated by Gram-positive cocci, specially *Staphylococcus aureus*, 74 (45.7%), and coagulase-negative staphylococci; rods of the family Enterobacteriaceae accounted for 14.1% and Gram-negative non-fermenting rods for 8.5% (Table 3).

**Table 3:** The most frequently isolated aetiological factors of SSI-D’s in 2009–2018

Pathogen	Total n(%)
*Staphylococcus aureus*	74 (45,4)
*Coagulase-negative staphylococci*	23 (14,1)
*Enterococcus faecium*	7 (4,3)
*Enterococcus faecalis*	4 (2,5)
*Streptococcus spp.*	1 (0,6)
*Escherichia coli*	7 (4,3)
*Enterobacter cloacae*	6 (3,7)
*Klebsiella pneumoniae*	9 (5,5)
*Citrobacter freundii*	1 (0,6)
*Acinetobacter baumannii*	8 (4,9)
*Proteus mirabilis*	3 (1,8)
*Pseudomonas aeruginosa*	2 (1,2)
*Serratia spp*	1 (0,6)
Not detected / not collected	17 (10,4)
Total	163 (100)

## Discussion

The results obtained in this study were divided into two time periods: years 2009–2013 (first period – before accreditation) and years 2014–2018 (second period – after accreditation). The actions implemented – a multimodal strategy – turned out not to be fully effective, since a significant fall in the incidence was obtained in only one category of treatments, i.e. both open and closed reductions of fractures, where the incidence was significantly decreased by 3 and 2.5 times. However, the fall in that single category was still a significant accomplishment for the investigated department, as these surgeries were most often performed. In the previous analyses by Wałaszek et al. [6], in 2008–2012, in the same department, FX incidence was 2.6–4.1%. In the literature, there are no other reports on the scale of this phenomenon in Polish trauma and orthopaedics wards. Furthermore, there are no reports from Europe on the incidence rate concerning infections associated with FX. In American NHSN research of 2006–2008 [10], the average SSI incidence associated with FX ranged from 1.1 to 3.4% depending on the presence of SSI risk factors, such as: duration of operation, ASA score, the degree of cleanliness of the operative field. A similar observation concerns CR procedures, for which in the studied department in 2008–2012, SSI incidence was 1.2–4.8% [6]. The nature of the procedure – no exposure of open tissues to external factors – and the fact that these procedures involved closed fractures, in which stabilization was introduced percutaneously, may indicate a close relationship between the occurrence of these infections and the moment of their implementation. It seems that the very labelling of these infections as a separate population in targeted surveillance directed the attention of the investigated department to the problem of SSI and resulted in reducing the number of SSIs. In the literature, data concerning the problem of SSI in such surgeries has not been touched upon.

On the other hand, the observed total incidence of 1% is a significantly good result considering previous Polish reports concerning SSI in orthopaedic surgery, e.g. in Sosnowiec, the SSI incidence was 6.6% [11], and 2.6% in Cracow [12]. However, multi-centre data are needed to give a more complete picture of the situation and allow us to draw comparisons. Rational inference is limited, given the lack of data on the SSI epidemiology in Polish trauma and orthopaedics wards, and considerable discrepancies in the epidemiological results. Therefore, it is recommended to implement a broad and unified HAI surveillance programme, including SSIs, which would involve a large number of entities, also in Poland.

A research on European Union countries conducted by the ECDC in 2008–2009 confirms the differences in the incidence rate between various countries after HPRO and KPRO procedures; e.g. for HPRO, the lowest rate was recorded in the UK and Lithuania (0.3–0.4%), and the highest one in Norway and Malta (2.8–3.8%) [13]. The discrepancies are probably associated with the sensitivity of the method, as the presumed high SSI detection in Norway, with the organization of work and the whole healthcare system, as well as with the flow of information between different participants of surveillance systems, which is connected with infection detection in post-discharge care. At the same time, the risk of exposure to SSI following HPRO and KPRO observed in the examined ward for several years reflects the expected level of risk, i.e. it is comparable to the average obtained in the European HAI-Net programme. Also, the microbial aetiology does not differ from the reports of other authors [14].

Unfortunately, the data presented are not so optimistic. Our attention is drawn to the dominance of one of the forms of SSI, that is, the lack of superficial infections, despite the fact that they should make up – in the case of HPRO and KPRO – around 50–60% of cases [14]. At this point, two hypotheses can be made, one suggesting SSI-D overdetection, that is, the tendency to classify cases incorrectly, the other indicating too low detection of superficial SSIs. Therefore, it is very likely that the real SSI incidence is even 2 times higher. The investigated hospital does not conduct any procedures with respect to process validation regularly, either in terms of the correctness of procedure performance or as regards the correctness of infection classification. In the authors’ opinion, it is the most important element concerning infection control that currently requires implementation in the study hospital.

An interesting observation, made possible by this analysis, can be done as regards patient demographics. A review of European data indicates that Polish patients are significantly younger (67 years) than the average patient population operated for HPRO and KPRO in Europe: median of 72 years. Also other Polish reports confirm this observation, in studies conducted in two Polish orthopaedic centres in 2005, the median ages were 68 and 67 years [15]. These results may suggest that inhabitants of other European countries enjoy better health than people in Poland, who require surgical intervention 5 years earlier, on average. This fact is even more disturbing when data from OECD from 2017 is taken into consideration, since the average waiting time for HPRO in Poland was 405 days, while, for example in the Netherlands, it is 42 days [16].

This retrospective study has some limitations. Firstly, the research involves only one centre. Secondly, in the period studied, despite participation in the multicenter programme, the surveillance of infection method was not validated, hence, its sensitivity is not known in this particular case.

## Conclusion

The introduction of multimodal and multidisciplinary SSI prevention and epidemiology programmes in 2014 has resulted in lower SSI incidence rates in some types of orthopaedic operations. This trend was most strongly visible following FX and CR fracture reductions. After HPRO, KPRO and other procedures, a stable, expected level of SSI incidence was maintained. The results described confirm the possibility of implementing an infection surveillance system also throughout Poland. Making this system stronger and encouraging its participants to make the results public can reinforce the regional and national surveillance systems. Especially combining the tasks of an infection control team with preparation for accreditation allows effective implementation of infection control practices. Such an approach, encompassing structural elements and indicators of infection prevention and control, integrating multimodal and multidisciplinary solutions, make it possible to strengthen the organizational culture resulting in the reduction of SSI risk. Problems associated with improving the hospital infection surveillance systems probably affect many other Polish trauma and orthopaedics wards, hence such active SSI surveillance should be adopted by other hospitals in Poland.

## Data Availability

The datasets generated or analysed during this study are available and can be accessed from Anna Rozanska (e-mail: a.rozanska@uj.edu.pl) on reasonable inquiry.
